# Distinguishing between “Function” and “Effect” in Genome Biology

**DOI:** 10.1093/gbe/evu098

**Published:** 2014-05-09

**Authors:** W. Ford Doolittle, Tyler D.P. Brunet, Stefan Linquist, T. Ryan Gregory

**Affiliations:** ^1^Department of Biochemistry and Molecular Biology, Dalhousie University, Halifax, NS, Canada; ^2^Department of Philosophy, University of Guelph, ON, Canada; ^3^Department of Integrative Biology, University of Guelph, ON, Canada

**Keywords:** causal role, selected effect, ENCODE project, functional genomics

## Abstract

Much confusion in genome biology results from conflation of possible meanings of the word “function.” We suggest that, in this connection, attention should be paid to evolutionary biologists and philosophers who have previously dealt with this problem. We need only decide that although all genomic structures have effects, only some of them should be said to have functions. Although it will very often be difficult or impossible to establish function (strictly defined), it should not automatically be assumed. We enjoin genomicists in particular to pay greater attention to parsing biological effects.

There is renewed debate among biologists about the meaning of “function.” Much of this has to do with the claim of ENCODE investigators to have at last disproven the 40-year-old notion that our genome is mostly informationally nonfunctional “junk” (ENCODE Project Consortium et al. [2012]; Graur et al. [2013]; Niu and Jiang [2013]; Eddy [2012, 2013]; Doolittle [2013]). To the extent that the controversy reflects disagreement about the meaning and proper use of words, a resolution is possible.

In the philosophy of biology, the two dominant formulations of “function” are causal role (CR) functionality and selected effect (SE) functionality (Brandon 2013). The former is ahistorical and simply addresses the way(s) in which a component contributes to a stated capacity of some predefined system of which it is a part: What it in fact does. A “system” could be any structural component (such as the heart or brain) or process (such as circulation of blood or cognition) recognized as coherent and biologically relevant by an investigator. By contrast, SE functionality is history dependent (etiological) and invokes explanations based on natural selection—that is, how that feature contributed to enhanced survival and reproduction now and/or in the past—in other words: Why it is there. This distinction is typically applied at the level of the whole organism or its genome: Effects at the intragenomic or super-organismal level are often neglected or assumed to be reducible to function at the genomic level. For simplicity, we adopt this focus here, although we believe that the CR/SE distinction and parsing similar to that sketched in figure 1 are also legitimately applicable at lower (intragenomic) and higher (population, species, and possibly clade) levels.

**Fig. 1.—:**
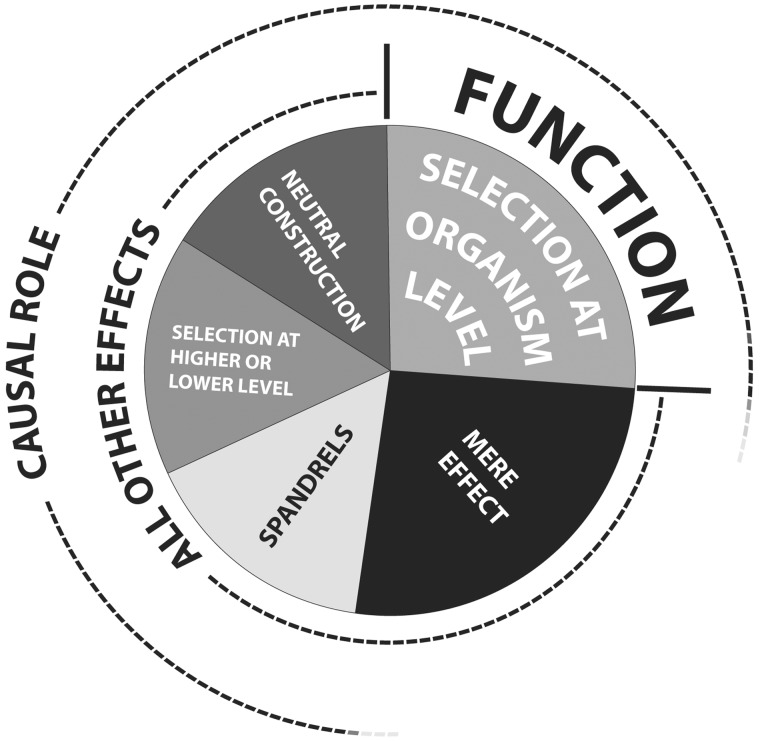
Types of effects. There is not a one-to-one mapping of effects to genetic elements, and sizes of the slices in this pie chart are arbitrary. Types of causal roles and SE function are considered here for the level of organisms, that which is usually implicit in genomic biology. “Mere effects” are consequences of the presence of a genetic element or sequence that might not generally be considered a “phenotype” at the organismal level, or to “contribute to the capacity of the system that contains it” in any biologically meaningful way. Being sensitive to restriction nucleases in vitro or templating its own replication in vivo, a property of every nucleotide, are examples. As phenotype becomes more significant or characteristically prominent at the organism level, the distinction between mere effects and spandrels becomes harder to make. The ability to support eyeglasses, clearly not an SE function, is nevertheless an important phenotypic consequence of noses, for instance. Indeed, the boundaries between all slices of this pie are negotiable, and depend on parameters that vary or other definitions about which there is no consensus. When population sizes are reduced, functions under weak selection might retain causal roles for some time, or quickly become mere effects. Products of the evolutionary ratchet called Constructive Neutral Evolution by definition arise neutrally but are maintained by purifying selection. Whether “selected effects” should be construed as embracing such elements has not been seriously addressed. And the effects of selfish elements at the organismal and species levels (negative and positive, respectively) might also be taken as spandrels at those levels.

SE functionality clearly pertains to a subset of components with CR functionality: No component can be selected and remain under selection unless it once made and still makes a contribution to the system that contains it. But many components, processes, or features that seem to be part of or characterize a system might well be accidental (biological “noise”), and not all “systems” that investigators might chose to define for study need to be products of natural selection. So to equate CR functionality with SE functionality, or conflate the two by not acknowledging such a distinction, is—whether admitted or not—panadaptationist. That is, it embodies the notion that natural selection is “so powerful and the constraints upon it so few that direct production of adaptation though its operation becomes the primary cause of nearly all organic form, function and behavior”—to quote Gould and Lewontin’s famous 1979 critique (Gould and Lewontin 1979).

In fact there are three ways in which a given trait may come to exert significant biological effects without having been shaped directly by natural selection operating at the level of organisms and their fitness-conferring genes (fig. 1). First, the effect considered may represent a side effect (a “spandrel” in Gould and Lewontin’s terminology) of other selected-for structures or processes. A favorite example of philosophers is the thumping noise made by the living heart, an invaluable aid in diagnosis but not the evolutionary reason that we have hearts. Molecular genetic examples could include the propensity of trinucleotide repeats to engender diseases or of heterochromatic regions to accumulate transposable elements—important effects, but hardly an explanation for their evolutionary origin or continued persistence within genomes.

Second, the trait or its effects could indeed be a product of natural selection, but at a level of organization lower (intragenomic) or higher (population or species) than the usual level of evolutionary explanation, namely organisms and their fitness-determining genes (Doolittle 1987; Gregory 2004). No one would consider the induction and replication of prophages to be the evolutionary “function” of bacterial cells; instead, it is well understood that there is selection at the level of the viruses themselves as well as among their bacterial hosts, so this would be a function of the prophages, not their hosts. Likewise, it would be odd to consider the harboring of nonviral retroelements to be a function of the human genome. These and other transposable elements are indeed products of selection, but at the intragenomic level rather than the organismal level, at least initially. Similarly, the wide prevalence (though probably not the origin) of sexual reproduction might best be explained by reference to selection above the organism level (i.e., among lineages). At every level at which selection might be said to operate, we imagine that the CR/SE distinction can be applied. Strictly speaking, some traits that are nonfunctional at the organism level might possess intragenomic or supra-organismal selected effects. Since the usual focus of functional discourse is on organisms, features selected positively or negatively at higher or lower levels but neutral (or negative) for organisms are considered to have only casual role functions for the purposes of figure 1.

Third, the trait and its effects may have arisen through neutral processes and not via selection at any level. That the neutral theory of molecular evolution explains most genomic primary sequence variation is widely accepted, even (perhaps paradoxically) by most who insist that our genome has no “junk.” If structurally complex elements of our genome can also arise by ratchet-like neutral processes, then SE functionality, insofar as it entails positive selection for a trait, should not automatically be assumed. One such ratchet has been called “Constructive Neutral Evolution” (Gray et al. 2010). In this process, one of two or more fortuitously interacting components “presuppresses” otherwise detrimental mutations that might occur in another, permitting such mutations to accumulate to a point of no return. A good example would be introns that initially functioned as ribozymes (protein-independent catalysts of their own excision) but have become dependent on proteinaceous “splicing factors,” initially only coincidentally associated but now obliged to coevolve. Another ratchet is genetic drift. By this mechanism, surprisingly complex molecular interactions will sometimes be fixed in small populations, even when disadvantageous (Lynch 2007).

What, then, of those cases that are not the result of natural selection on the trait and its biological contribution (selected effects, or as we argue here, functions sensu stricto), do not represent biologically important “side effects” of other elements under selection (spandrels), have not resulted from natural selection at lower or higher levels (parasitic DNA or traits fixed by selection operating among lineages), have not been built up by constructive but neutral processes, and play no obvious role in any nonarbitrarily constrained predefined system? These are “mere effects,” without significant biological consequence, though they may be useful to biologists. The sensitivity of certain DNA sequences to certain restriction endonucleases would be an obvious example, as would the ability of proteins to form crystals (unless that is their normal biological state, as for lens crystallins) or the melting temperature of the DNA double helix (except for extreme thermophiles). More directly observable examples might be the color of blood (aside from its possible importance in blushing) or colony formation on plates of bacteria that do not in nature form colonies. One might also consider as “mere effects” elements that play a causal role in “predefined” systems that are obviously themselves not products of selection, for all they may be of great significance to biologists. For example, many studies impute “function” to cell components or activities involved in the initiation and progression of diseases. Thus, many readers might be comfortable with statements such as “gene X functions in the progression of disease Y.” But most would balk at a claim that “the function of gene X is to contribute to disease Y.”

Causal roles are easiest to infer experimentally, and much of functional genomics and molecular genetics surely has such inference as its immediate goal. However, not all causal roles that can be conceived or demonstrated empirically are biologically meaningful. Indeed, it has been well acknowledged by philosophers who favor the CR concept that there is a danger in defining the “system” and its “capacities” too broadly, such that reported causal roles become entirely investigator-dependent, even stretching to include what we are calling “mere effects” (Elliott et al. 2014). For this reason, potential SE functions are often tacitly sought and assumed to exist by researchers when deciding which causal roles among the (possibly infinite) list of options are worthy of investigation. The more complex the causal role the more appealing this assumption—but still, the complex rhythmic noise made by the heart provides a straightforward cautionary example, as might the correlation between transcription factor-binding site numbers and noncoding DNA among genomes (Ruths and Nakleh 2012). Pro-CR philosophers, for their part, have tended to defer to the common sense of biologists in this regard, confident that they are unlikely to abuse the concept of CR functionality in their studies. Sadly, the extremely loose CR definition used by ENCODE as the basis of their claims that more than 80% of the human genome exhibits a “biochemical function” shows this confidence to have been misplaced.

There exist a great many effects in biology—consequences of the presence or activity of structures or processes. Many of these perform casual roles of considerable biological interest, regardless of the explanations(s) for their evolutionary origin and continued persistence. CR reasoning is the bread and butter of developmental biology, disease research and genetic manipulation, and an invaluable tool for the preliminary identification of candidates for SE functionality. But we really do not know what fraction of CR-identified traits are SE functional, or at what level, and the role of other sources of complexity, such as neutral evolutionary ratchets, remains undetermined. It seems unnecessarily misleading to assume that CR methods alone can establish “function” in a biologically meaningful sense of the word.

Historical causation is of course often very difficult to infer and impossible to prove beyond all doubt: We can only marshal more and more evidence. But were SE functionality to be dismissed as unproveable evolutionary speculation, we would lose an invaluable distinction and conceptual tool, and the danger that claims about CR relationships will be taken to imply selective evolutionary histories where none exist would be intensified. Worse, to abandon the distinction between selected and unselected effects, difficult as it might be to draw in practice, would be to give up on what makes biology unique and comprehensible as a science. So let us keep “function” tied to selective history while valuing and pursuing CR-based studies of phenotypic “effects,” recognizing that they are noncommittal as to real biological function in the strict sense. Thus, in many contexts where “function” has been used as a noun, “effect,” “consequence,” or “activity” would be more appropriately neutral, and “casual role” (or simply “role”) would remain fully accurate. And if, as we anticipate, this recommendation is not immediately or widely accepted, at least we might hope for expanded and more nuanced discussion of the meaning of “function” when it is at issue. Conflation is the enemy of understanding.

## Note Added in Proof

In a response to previous critiques which appeared as this Perspective was in final revision, ENCODE investigators admit to some difficulties around defining function (Kellis et al. 2014). Remarkably, however, these authors focus on reconciling "the strengths and limitations of biochemical, evolutionary, and genetic approaches for defining functional DNA segments" but avoid dealing with the central conceptual issue, which is the problematic nature of "function" itself. A simple folk-philosophical dismissal of this issue leaves the confusion over "junk DNA" unresolved.
